# Transcatheter and intraoperative device closure and surgical repair for atrial septal defect

**DOI:** 10.1186/s13019-019-0957-0

**Published:** 2019-07-19

**Authors:** Han-Fan Qiu, Qiang Chen, Zhi-Nuan Hong, Liang-Wan Chen, Xue-Shan Huang

**Affiliations:** Department of Cardiovascular Surgery, Union Hospital, Fujian Medical University, Fuzhou, 350001 People’s Republic of China

**Keywords:** Surgery, Congenital heart diseases, Atrial septal defect, Devices closure

## Abstract

**Background:**

Transcatheter and intraoperative device closure for atrial septal defect (ASD) are widely applied to reduce the incision size and the potential for injury during cardiopulmonary bypass (CPB) in conventional surgical repair. No studies had been conducted to compare the safety and efficiency of these three treatments.

**Methods:**

From January 2018 to April 2018, 87 patients with an isolated ASD who had undergone transcatheter device closure (*n* = 45), intraoperative device closure (*n* = 22) and surgical repair (*n* = 20) were retrospectively reviewed and further analyzed to compare these three treatments.

**Results:**

The successful closure rate was similar in the three groups. There was a significant difference in aortic cross-clamping time, CPB duration and operative time between the surgical group and the device groups. The length of intensive care unit stay, postoperative mechanical ventilation time and length of hospital stay were shorter in the two device groups than in the surgical group. The incision was the most extended in the surgical group. Regarding major adverse events, no significant differences were found among the three groups.

**Conclusions:**

Transcatheter and intraoperative device closure and surgical repair for ASD are all safe and effective. Considering their respective disadvantages and advantages, the transcatheter approach may be the first choice for an isolated secundum ASD, the intraoperative approach may be the second choice, and surgical repair may be the last resort.

## Introduction

Atrial septal defect (ASD) is a common cardiac malformation and accounts for approximately 8 to 10% of cases of congenital heart disease (CHD) [1]. Patients with an isolated ASD may not show symptoms during infancy or childhood, and thus, the diagnosis may not be made until adolescence or adulthood. The treatment of ASD is generally recommended because increased pulmonary blood flow may lead to pulmonary hypertension. Surgical repair for ASD under cardiopulmonary bypass (CPB) has been the standard treatment [2, 3]. With the widespread use and development of various occluders, transcatheter device closure for ASD has gradually gained popularity and served as an alternative to conventional surgical repair [4, 5]. Recently, intraoperative device closure for ASD has been widely performed in mainland China [6, 7]. In this study, above three treatments were applied for patients with an isolated secundum ASD and reviewed the relevant literature. Few comparative studies of these three approaches have been performed. Therefore, we report our experience with and compare these three treatments.

## Materials and treatments

Before choosing the treatment, all guardians of the patients were informed of the indications, contraindications, advantages, disadvantages and specific risks associated with each treatment. In particular, the surgical approach requires CPB and an incision, foreign bodies are implanted in the treatments, and the occluder might become dislodged in the device groups. In addition, while there is no incision in the transcatheter approach, there may be the potential for vascular injury and X-ray exposure, among others. The guardians considered their own condition and willingness adequately and communicated with the doctors; then, they chose the treatment suitable for their patient and provided written informed consent.

We reviewed the medical records of a total of 87 patients who had undergone ASD closure in our institution (Union Hospital, Fujian Medical University) from January 2018 to April 2018 in this study. The patients were divided into three groups according to their guardians’ willingness to choose different treatment options. There were 45 patients in group A (transcatheter device closure), 22 patients in group B (intraoperative device closure) and 20 patients in group C (surgical repair). The patients’ clinical characteristics are shown in Table 1. Regarding sex, age, and body weight, no significant differences were found among these three groups. Routine clinical examinations were performed before the procedure, including a standard lead electrocardiogram, a chest X-ray examination, and blood and biochemical tests. All patients in the present study were diagnosed with an isolated secundum ASD by transthoracic echocardiography (TTE). The inclusion criteria were a secundum ASD without other cardiac malformations and significant left-to-right shunting and atrial overloading, with or without mild pulmonary hypertension. Patients with idiopathic pulmonary hypertension, a deficiency in part of the rim, or another concomitant congenital heart disease requiring surgical intervention were excluded from this study [7, 8]. We defined successful ASD closure as the lack of a sizeable residual shunt (< 2 mm) as evaluated by postoperative TTE.

**Table 1:** Preoperative data comparison among three groups of patients

Item	Group A	Group B	Group C	*P*
*N*	45	22	20	
Age (year)	4.7 ± 2.3	4.4 ± 2.8	4.3 ± 3.1	0.983
Gender(M/F)	24/21	12/10	10/10	0.958
Weight (kg)	16.3 ± 8.2	17.5 ± 7.8	16.7 ± 8.5	0.938
Size of ASD (mm)	18.4 ± 6.2	20.8 ± 5.3	22.1 ± 8.6	0.804
Pulmonary hypertension (mmhg)	26.5 ± 5.6	32.7 ± 5.2	31.4 ± 4.2	0.348
Cardiothoracic ratio	0.51 ± 0.06	0.51 ± 0.08	0.56 ± 0.05	0.579

### ASD Occluder device

In group A, the Amplatzer ASD device and a domestic ASD occluder (Shan Dong Visee Medical Apparatus Co., Ltd., China) were used, and a standard transfemoral approach was adopted. In group B, the ASD occluder used was also manufactured by Shan Dong Visee Medical Apparatus Co., Ltd., China, which we have described previously was the same as that in group A. The domestic occluder was made from an alloy of nickel and titanium. Other components consisted of a sheath and a pushing rod. The occluder was selected according to the TTE evaluation as the maximum defect diameter plus 2–6 mm in group B [7].

### Operative technique

#### Transcatheter device occlusion (group a)

The procedure has been previously described and detailed in many papers, and it was performed in the catheter laboratory/operating room under local/general anesthesia following TTE guidelines (with/without X-ray guidance) [4, 5, 9, 10]. Catheterization was accomplished through the femoral vein, and the defect was bypassed with the guidewire. The ASD diameter was assessed by TTE and/or angiography, and a corresponding occluder selected was 1–2 mm larger than the obtained measurement. The occluder was released with TTE guidance.

#### Intraoperative device closure (group B)

Intraoperative device closure was performed under general anesthesia. Patients were placed in a supine position and then exposed the chest with the right thorax elevated approximately 30°. TTE/transesophageal echocardiography (TEE) was applied to assess the situation and the ASD shape and size, especially its relationship with the circumferential cardiac structure. A minimal incision was made via right anterior submammary thoracotomy (approximately 3 cm). The pericardium was opened and suspended to expose the right atrium. Through this approach, a “purse-string” suture approximately 1.5–2.0 cm in diameter was stitched in the right atrium. Heparin was given intravenously (1 mg/kg body weight) before the operation. The occluder was loaded into the delivery sheath, and then an incision was made in the “purse-string” suture. The delivery sheath was inserted into the right atrium and then advanced it through the ASD into the left atrium guided by TTE/TEE. The left and right discs were carefully deployed in turn by pushing the rod to close the ASD [6, 7]. Oral dipyridamole or aspirin was administered (1–2 mg/kg) for 3–6 months as an anticoagulant.

#### ASD surgical repair via median sternotomy (group C)

Three incisions were used, one for median sternotomy, right anterolateral thoracotomy, and right vertical infra-axillary thoracotomy, according to the patient’s height, weight, sex, and needs. A pericardial patch was used in all cases. All patients required CPB in this group.

### Statistical analysis

Continuous variables with a normal distribution are expressed as x ± s, and T-Test or ANOVA was used to compare continuous variables, while the χ2 or Fisher’s test was used for categorical variables. A *p* value< 0.05 was defined as indicating statistical significance.

## Results

The rate of successful device closure was 97.7% immediately after the operation and 100% in the one year’s follow-up appointment in group A, which was not significantly different from that in group B (95.6 and 100%, respectively). One patient in group A was converted to surgical repair due to occluder dislodgement during the procedure, this patient was excluded from the corresponding follow-up data and was also not enrolled as part of the surgical group. Surgical repair was performed in group C, and was achieved successful closure in all patients.

Regarding the postoperative length of hospital stay, no significant differences were found between group A and group B. Only a few patients in the laboratory in group A needed fluoroscopy. The postoperative pulmonary infection rate was higher in group B and group C than in group A. Patients in group C required the longest operative time, mechanical ventilation time, postoperative hospital stay and ICU stay. Meanwhile, patients in this group also needed the most blood product. Cardiopulmonary bypass with/without aortic cross-clamping was only required in the surgical group. The length of the different incisions was nearly 6–9 cm in the surgical group, while the incision length in group B was approximately 2–3 cm (Table 2). The occurrence rate of transient perioperative arrhythmia among these three procedures was similar. There were no instances of death, low cardiac output syndrome, atrioventricular block, other relative organ dysfunction, other comparable complications requiring reoperation, or cerebrovascular events recorded in the present study (Table 3).

**Table 2:** Intraoperative and post-operative data comparison among three groups

Item	Group A	Group B	Group C	*P*
Occluder size (mm)	22.3 ± 4.2	26.5 ± 6.4	/	0.569
Operative time (min)	46.2 ± 9.5	34.5 ± 10.3	75.6 ± 18.8	0.024
Cardiopulmonary bypass (min)	/	/	36.6 ± 8.2	
Fluoroscopic time (min)	1.2 ± 1.1	/	/	
Mechanical ventilation time(h)	/	3.9 ± 1.7	10.6 ± 7.2	0.045
Intensive care unit time(h)	/	5.8 ± 1.8	12.2 ± 5.1	0.028
Drainage (ml)	/	21.6 ± 9.5	56.6 ± 27.5	0.038
Blood transfusion (ml)	0	0	245 ± 35	0.000
The incision length (cm)	/	2.2 ± 1.3	7.6 ± 1.1	0.013
Postoperative hospital stay(d)	2.2 ± 1.1	3.2 ± 1.7	6.6 ± 4.0	0.047

**Table 3:** Post-operative complications comparison among three groups

Item	Group A	Group B	Group C	*P*
Significant Residual shunt	0	0	0	
Pulmonary infection	0	2	5	0.001
Surgical wound bad healing	0	1	2	
Pneumothorax	0	1	0	
Thoracic deformity	0	0	1	
Pericardial effusion	0	0	0	
Pleural effusion	0	1	0	
Transient Arrhythmia	11	5	3	0.776
Device embolization	1	0	/	
Hematoma at access site	0	0	0	

The follow-up period was 12–15 months. All patients underwent a physical examination, electrocardiography, and TTE. No severe complications, such as sudden death, cerebral embolism, cardiac perforation, aortic laceration, cardiac valve distortion, endocarditis, or malignant arrhythmia, were observed in the follow-up period.

## Discussion

Conventional surgical repair under CPB via the median sternotomy approach is the standard treatment for ASD [2, 3]. Other surgical incisions, including right anterolateral thoracotomy and right axillary thoracotomy, are used to obtain better cosmetic results [11–14]. However, these surgical treatments are all associated with operative trauma, the potential risk of CPB, visible scar formation, postoperative discomfort and the possible need for blood transfusion. Transcatheter device closure for ASD has been widely used with satisfactory early and mid-term results. With the development of technology and devices, transcatheter device closure for ASD has gradually become the first choice in select patients [15–17]. Intraoperative device closure for ASD has also been widely applied in China and has the advantages of no X-ray exposure, ease of operation, ease of learning and mastering, and a relatively minimal cosmetic incision [6, 7]. From previous studies showed that the three treatments were all safe and effective for ASD closure with their own advantages and disadvantages.

There has been extensive sample data analysis for these three treatments for ASD closure, and studies comparing transcatheter or intraoperative device closure and surgical repair have been reported [18–21]. Berger F. and his coworkers published a series of 61 patients who underwent ASD surgical repair at a median age of 20 years (0.5–74 years) and 61 patients who underwent transcatheter Amplatzer device closure for ASD at a median age of 12 years (0.8–77.7 years). Their results showed that similar complete closure and complication rates in both groups, but the duration of hospital stay was shorter with less morbidity in the device group. Thus, they prefer the deployment of an Amplatzer septal occluder to surgical repair whenever possible [22]. Qiang Chen and his colleagues previously reported on 252 patients who underwent secundum ASD closure, including 182 patients who underwent intraoperative device closure and 72 patients who underwent surgical repair with a follow-up period ranged from 1 to 5 years. The results showed that intraoperative device closure for ASD was a safe and feasible technique in select patients. This approach has the advantages of a lower cost, better cosmetic results, and less trauma than surgical closure [7]. As there have not been studies focusing on comparing these three treatments, we examined the three treatments and reported our experience.

Compared with the surgical group, the two device groups had similar rates of success and complications and faster recovery in terms of the operative time and postoperative hospital stay. Both device approaches were less invasive than the surgical approach and showed satisfactory early and mid-term results. No patients required a drainage tube, blood transfusion, ICU stay, or mechanical ventilation in group A. In this study, one patient from group A underwent surgical repair due to occluder dislodgement back into the right atrium in the operating room. During the operation, the inferior rim of the ASD was confirmed to be deficient in this case. Additionally, the mortality rate of surgical closure for ASD is close to zero, and significant morbidity is rare in our institution.

Compared with approach A, approach B has broader indications with fewer limitations based on weight, age, and ASD size. Although recent reports have shown that operators tend to have more experience in large ASD closure using the transcatheter device approach, it is still a technical challenge for most operators, especially for those with a lack of experience [23, 24]. Lairakdomrong K. et al. reported that 30 patients with an ASD size equal to or greater than 30 mm and 32 patients with an ASD size less than 30 mm all underwent transcatheter closure. The complication rate was higher in patients with a large ASD and treated with a device ≥30 mm, especially during the learning curve period [23]. Fraisse A. et al. emphasized that patients with large ASDs (> 38 mm) and defects with deficient rims are usually referred for surgical closure rather than transcatheter closure [25]. An intraoperative approach could provide satisfactory clinical results and eliminate the risk of occluder dislodgement from large ASDs [26]. Hongxin L. and his coworkers reported on 67 patients with a large secundum ASD, approximately of which had one short rim, who underwent intraoperative device closure; they concluded that their method was a safe and feasible technique for closing large ASDs [27].

In our opinion, we think that the indications for approach B could be broader, for the following reasons. First, the shorter delivery system offers a relatively perpendicular angle with respect to the atrial septum, and the learning curve is short, which could allow for easier manipulation. Second, the operator could perform a direct push-pull test to check and confirm the stability of the occluder. Third, the percutaneous approach is sometimes limited by a patient’s weight and peripheral vascular access. Fourth, a ‘left atrium-occluder-right atrium’ suture can be placed via the incision to fix the occluder in the intraoperative approach, which is especially suitable for large ASDs with a deficient rim [21]. Additionally, both surgeons and patients can avoid X-ray exposure because TTE/TEE is used to guide the entire process in approach B.

According to previous reports and clinical observations, occluder dislodgement or embolization is a rare but severe complication of device closure procedures [28]. Fortunately, only one patient in group A suffered occluder dislodgement in the perioperative period and underwent surgical repair. A residual shunt is a common complication of device closure for ASD, especially for patients with a large ASD. Trivial or small residual shunts (< 2 mm) occurring immediately after the occluder deployment can be ignored since they usually disappear during the follow-up period [26, 27]. This first residual shunt occurred in the free links between the occluder and the rim of the defect or the occluder itself. Endothelialization would cover the surface of the occluder and then close the residual shunt.

Reports have shown that radiation exposure, even at low doses, could increase the risk of cancer [29]. It is essential to avoid unnecessary radiation exposure, especially in special populations. Considering the limited medical resources in our circumstance, we have mainly performed intraoperative device closure for ASD over the past 5–10 years [7]. Recently, we began to use TTE as the only guiding tool in transcatheter device closure for ASD, and we have also reported our initial experience with this method [9].

In summary, for select patients with a secundum ASD who would be suitable for device closure, transcatheter device closure completely guided by TTE can be used as the first choice, followed by intraoperative device closure as the second choice and surgical repair as the last resort. In some patients with insufficient rims who may be not suitable for transcatheter device closure, intraoperative device closure can be as the first choice. However, surgery is still preferred for patients who reject foreign implants and are unsuitable for device occlusion. In addition to communication with the guardians, individualized treatment options are also important. It is worth mentioning that the operating room can be a “one-stop shop” for ASD treatment, in which these three treatments would be easy to generalize and apply.

This study is limited by its retrospective nature. There may be selection bias in the collection of data, which may have reduced the reliability of the conclusions, But it still has some clinical significance. This was a single-institution study, and a multicenter study is necessary to obtain further evidence. Furthermore, more extended follow-up periods are required in subsequent studies.

## Conclusion

Transcatheter and intraoperative device closure and surgical repair for ASD have been shown to be safe and effective. Considering their respective disadvantages and advantages, we recommend the following sequence of treatment options: a transcatheter approach, an intraoperative approach, and surgical repair.

## Data Availability

Data sharing not applicable to this article as no data sets were generated or analyzed during the current study.

## Abbreviations

ASD: atrial septal defects
CHD: congenital heart disease
CP: cardiopulmonary bypass
TEE: transesophageal echocardiography
TTE: transthoracic echocardiography
